# A constructive model for collective intelligence

**DOI:** 10.1093/nsr/nwaa092

**Published:** 2020-05-07

**Authors:** Wei Zhang, Hong Mei

**Affiliations:** Key Laboratory of High Confidence Software Technology (Peking University), Ministry of Education, China; Institute of Software, Department of Computer Science and Technology, Peking University, China; Key Laboratory of High Confidence Software Technology (Peking University), Ministry of Education, China; Institute of Software, Department of Computer Science and Technology, Peking University, China

## Abstract

Natural phenomena of collective intelligence (CI) occurring in physical space show a potential approach to effective large-scale human collaboration in cyberspace. Based on existing explanatory understanding of CI, this perspective proposes a constructive model for building artificial CI systems, i.e., problem-oriented CI phenomena with AI-powered information integration and feedback.

For a long time, scientists have observed seemingly paradoxical phenomena in many kinds of social insect: each individual either does not have, or has very limited, intelligence. However, a collective of them often shows much higher intelligence than individuals. Intelligence exhibited at the collective level of a group of individuals is called *swarm intelligence* or *collective intelligence* (CI). Two valuable properties are observed from those natural CI phenomena in physical space: the *amplification effect* on individual intelligence, and the *scalability* to the number of participating individuals. In essence, CI results from effective large-scale collaboration of autonomous individuals.

**Figure 1.**
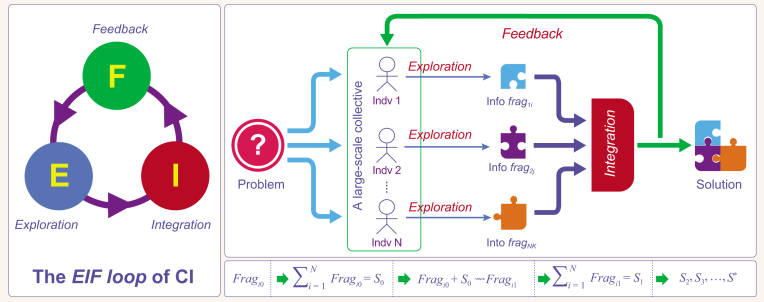
A constructive model for CI, which manifests itself as a continuously executing loop, consisting of three activities: *exploration*, *integration* and *feedback*. We name this model the *EIF loop* or simply *EIFL*.

Natural CI phenomena in physical space show a potential approach to effective large-scale human collaboration in cyberspace, since it is much more economical to assemble a large group of people in cyberspace than in physical space. If the mechanism embodied in CI is applied to a large group of cyberspace-connected people, and the amplification effect on individual intelligence emerges, we believe it will unleash a powerful force of human societies and facilitate human civilization in reaching a brand-new level [1].

## CURRENT PRACTICE AND RESEARCH

Many CI phenomena have been observed in physical space, involving lower organisms/animals, or even human beings. Most of these phenomena result from long-term natural selection. Besides CI phenomena in physical space, many insightful human-involved CI phenomena or systems have appeared in cyberspace, manifesting innovative problem-solving methods in various domains. Some of these phenomena/systems are results of long-time socio-technical co-evolution, and some of them are carefully designed to resolve specific problems. For example, in the domain of software engineering, after decades of evolution, open source software development [2] has become an important kind of socio-technical phenomenon, in which many geographically distributed developers collaborate with each other through the Internet and have successfully developed various kinds of complex software systems. In the domain of problem solving, the unanimous.ai system [3] provides an online platform that enables a large group of people to collectively give answers to multiple-choice questions, showing high accuracy when solving prediction or decision-making problems in many real scenarios. In the domain of biological research, the EteRNA system [4] is a multi-player online game, which has successfully engaged a large number of non-scientists in solving complex protein structure problems.

Current research on CI focuses largely on explanatory aspects of CI, that is, how to explain the occurrence of observed CI phenomena. Most current practices on CI are case-by-case or *adhoc*: some focus on duplicating and modulating natural CI phenomena, to validate their explanatory understanding of CI, and some focus on *adhoc* construction of human-involved CI systems, to resolve specific problems based on large-scale collaboration. There is still a big gap between explanatory understanding of CI phenomena and construction of useful CI systems. A typical example is the concept of *stigmergy*, which was originally coined to explain CI phenomena in social insects [5] and also gradually shows applicability in analyzing and explaining humaninvolved CI phenomena [6,7]. We do believe that stigmergy provides an insightful explanatory model of CI, behaving well in *post hoc* analysis of existing CI phenomena. However, it is still unclear to what degree stigmergy can be used to direct construction of CI systems.

The main problem in current research is that those explanatory understandings of CI phenomena cannot effectively satisfy the requirements of constructing CI systems to cope with complex problems in real situations; all those well-known human-designed CI systems are not constructed under guidance of mature theories of CI. Resolving this problem calls for theories or methods that explicitly touch the constructive aspect of CI, i.e. how to construct CI systems in disciplined ways.

## A CONSTRUCTIVE MODEL

In order to explore constructive theories of CI, it is natural to take a problem-oriented viewpoint: any CI phenomenon aims to solve a problem. This viewpoint sets up motivation for constructing CI systems. In particular, we treat the mechanism embodied in CI as a way to solve complex problems based on large-scale collaboration.

Based on the problem-oriented viewpoint, we propose a constructive model for CI (see Fig. 1). The essence of CI is a loop consisting of three activities: exploration, integration and feedback. Given a problem and a collective of individuals, the problem can be resolved collectively in the following way: in *exploration*, each individual freely explores the solution space and contributes a set of information fragments valuable for resolving the problem; in *integration*, all these information fragments are merged/integrated together, resulting in a set of well-structured information fragments or a set of partial solutions to the problem; and in *feedback*, results of integration are fed back to individuals, stimulating them to improve the sets of information fragments they have contributed. This *exploration*-*integration*-*feedback loop* (EIFL) will continuously execute until an acceptable solution is found.

EIFL-enabled problem solving embodies itself in an incremental, iterative and parallel process: whenever individuals contribute new information fragments, the fragments will be merged with existing fragments, and then updated merging results will be fed back to the collective. It usually takes many iterations to obtain an acceptable solution, and different individuals conduct exploration in parallel, without explicit dependence on each other.

The EIFL model is conceptual, because it abstracts away many important details in real CI phenomena, including: (i) how the information fragments (i.e. output of exploration) manifest themselves; (ii) how these fragments are integrated; (iii) how integrated results are fed back to the collective; and (iv) which are actors of integration and feedback. Different CI phenomena are distinguished by different implementations and combinations of these details.

**Figure 2.**
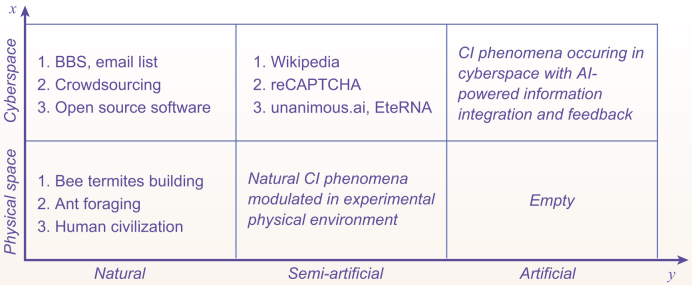
A two-dimensional classification of CI phenomena.

Furthermore, we propose a classification of CI phenomena in two dimensions (see Fig. 2). More dimensions can be introduced, if necessary (e.g. a possible dimension is the type of individuals, and the possible values in this dimension include natural creature, artificial agent or hybrid). Dimension $x$ relates to which space CI phenomena occur, consisting of two values: physical space and cyberspace. Dimension $y$ is the fourth detail mentioned above, i.e. actors of integration and feedback, characterized by three values: natural, artificial and semi-artificial. Here, the word ‘artificial’ has the same meaning as in ‘AI’. Dimension y focuses on the degree to which the integration and feedback activities in CI are carried out by AI techniques. It should be pointed out, when considering CI phenomena in cyberspace, that we view the capabilities of information storage and transmission provided by cyberspace as natural. In a natural CI phenomenon, integration and feedback happen naturally, without involvement of artificial machines (i.e. human-made automated machines/algorithms); in an artificial one, the two activities are carried out completely by artificial machines; and in a semi-artificial one, the two activities are accomplished by a combination of artificial machines and other components (e.g. physical laws or human individuals).

In the following, we re-investigate some representative CI phenomena using the EIFL model to show more details of EIFL.

Collective foraging behavior in ant colonies is a kind of *natural physical* CI phenomenon, in which ants find and maintain a trail between the nest and a food source collectively, until the food source is exhausted. In this phenomenon, (i) information fragments are represented as pheromones in the physical environment left by ants when carrying food back to the nest; (ii) pheromones left by ants at the same locations are merged together, resulting in an increased density of pheromones in those locations, and meanwhile, the density of pheromones is gradually decreased because of evaporation effect; (iii) pheromones in the environment are fed back to individuals according to their location (as a result, individuals perceive density of pheromones at their current location, stimulating them to follow the trail of pheromones and find the food source); (iv) physical laws are actors of integration and feedback.

Traditional human knowledge discovery is a kind of *physical* CI phenomena, in which many kinds of EIFL exist according to how knowledge/information is recorded in physical space. One such loop uses books as the vehicle for information recording, in which information fragments of an issue are observed and organized into a book by the author, and then the publisher prints the book and distributes its copies to different places, where a person may buy the book and improve her/his understanding of the issue from information in the book. In this example, information fragments (i) are embodied in different practices of an issue, (ii) are transformed into text and integrated into a book, and (iii) are fed back to the collective through book distribution. Actors of integration and feedback are individuals with different roles (i.e. the author and publisher), as well as possible human-made machines (e.g. printing machines). Depending on whether machines are involved, traditional human knowledge discovery can be *natural* or *semi-artificial*.

The EIFL model also gives the causation as to why human society rarely uses physical human CI to resolve problems having stringent time and budget constraints, and why various human CI phenomena are gradually emerging in cyberspace [8]. For the first ‘why’, there are three causes: (i) high cost of assembling a large group of people in physical space; (ii) low speed of information transmission in physical space; and (iii) dependence on a small elite to integrate large-scale information fragments and low efficiency of integration. For the second ‘why’, a cause is that the first ‘why's’ former two causes just disappear in cyberspace.

The unanimous.ai system [3] is a cyber CI system that enables a large group of people to collectively solve multiple-choice questions. The question-solving process embodies an EIFL: (i) each individual shows her/his opinion by placing a virtual magnet at an appropriate position relative to a virtual puck (i.e. *exploration*); (ii) all forces exerted on the puck from magnets are integrated into a single force, making the puck move in a specific direction (i.e. *integration*); (iii) the puck's movement is observed by all individuals (i.e. *feedback*), stimulating them to adjust positions of their magnets accordingly (i.e. a new iteration of *exploration*); (iv) actors of integration and feedback involve both algorithms specific for unanimous.ai and the natural information transmission capability of cyberspace. Therefore, the unanimous.ai system is *semi-artificial*.

The EteRNA system [4] is a cyber CI system that engages non-scientists in solving protein structure problems. An EIFL also exists in this system: (i) players design their solutions in their own workspaces (i.e. *exploration*); (ii) players review and vote for the best solutions, and a set of top-voted solutions are synthesized and verified by chemical measurements (i.e. *integration*); (iii) results are published online (i.e. *feedback*), stimulating players to start the next iteration of problem solving; and (iv) the actors of integration and feedback involve humans, algorithms specific for EteRNA, and the natural information transmission of cyberspace. Therefore, the EteRNA system is *semi-artificial*.

In addition, it should be noted that the category of artificial physical CI phenomena is empty. It is impossible to construct pure artificial CI phenomena in physical space.

## A POSSIBLE FUTURE

We believe that artificial or semi-artificial human CI phenomena will become ubiquitous in cyberspace in the next few decades, to resolve real complex problems from different domains. In particular, for different types of problem, specific methods and algorithms for information representation, integration and feedback will be developed to support effective large-scale human collaboration and problem solving. Information fragments will be integrated by considering more and more semantic relevance, and integrated information will be fed back in more proactive and personalized manners in accordance with each individual's capability and characteristic, so that each individual receives the right set of information from the collective to unleash the individual's potential in information exploring and problem solving, resulting in more diverse exploration of individuals and more effective exploration of the collective. We have named this type of phenomena ACI (artificial CI). ACI is problem-oriented CI occurring in cyberspace, with AI-powered information integration and feedback.

In order to facilitate this future's realizing, besides non-technical issues, three technical problems should be investigated sufficiently.

- How to quantitatively evaluate whether or when CI emerges from large-scale collaboration and then the degree of the emerged CI. Solutions to this problem will provide objective functions to guide the exploration of different implementations of EIFL.
- Whether general or domain-oriented mechanisms can be found for large-scale information representation, integration and feedback, so that these mechanisms can be reused when building CI systems. Solutions to this problem are closely related to existing AI research, including knowledge graphs, recommendation algorithms, etc. Currently, we are exploring graph-based approaches to this problem in the fields of software development [9] and jigsaw puzzles [10]: the former studies the value of graph-based information representation in program merging; the latter focuses on the resolving of EIFL-based jigsaw puzzles, and can be experienced at https://www.pintu.fun.
- Whether a formal theory can be developed for human-dominating evolution-based problem solving. Solutions to this problem will clarify the limitation of CI, afford theoretical assurance of CI systems’ convergence, efficiency and effectiveness, and also provide insights for how to manage the whole process of CI-based problem solving.

As we have observed, the development of human civilization is a continuous process of problem experiencing and resolving. In order to resolve various problems it has been confronted with, the human species has depended much on pervasive collaboration between human individuals in physical space. Based on this success, the further development of human civilization would closely relate to how human collaboration could evolve itself into more effective forms in accordance with a dramatically changing technical environment, i.e. Internet-based cyberspace's emerging and flourishing.

Cyber-based CI shows a potential way of effective large-scale human collaboration. A lot of problems could be resolved by ACI systems with increased efficiency and effectiveness, if appropriate theories and methods for CI and construction of ACI systems were developed. The EIFL model proposed in this article reflects our efforts to facilitate the realization of this brighter future of human collaboration. We conceptualize EIFL as a constructive model for CI, and a refinement of the existing explanatory understanding of CI. We hope the EIFL model could contribute to more fundamental theories of CI, and ACI systems could really show their value in the resolving of complex problems and in the further development of human civilization.
